# Solid-State Structural Properties of Alloxazine Determined from Powder XRD Data in Conjunction with DFT-D Calculations and Solid-State NMR Spectroscopy: Unraveling the Tautomeric Identity and Pathways for Tautomeric Interconversion

**DOI:** 10.1021/acs.cgd.1c01114

**Published:** 2021-11-22

**Authors:** Christopher J. H. Smalley, Andrew J. Logsdail, Colan E. Hughes, Dinu Iuga, Mark T. Young, Kenneth D. M. Harris

**Affiliations:** † School of Chemistry, Cardiff University, Park Place, Cardiff CF10 3AT, Wales, United Kingdom; ‡ Cardiff Catalysis Institute, School of Chemistry, Cardiff University, Park Place, Cardiff CF10 3AT, Wales, United Kingdom; § Department of Physics, University of Warwick, Coventry CV4 7AL, England, United Kingdom; ∥ School of Biosciences, Cardiff University, Cardiff CF10 3AX, Wales, United Kingdom

## Abstract

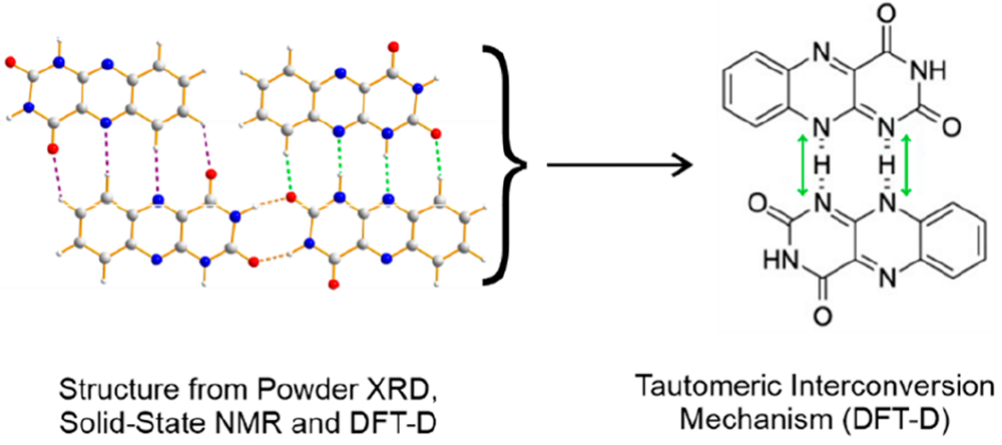

We report the solid-state structural properties of alloxazine, a tricyclic ring system found in many biologically important molecules, with structure determination carried out directly from powder X-ray diffraction (XRD) data. As the crystal structures containing the alloxazine and isoalloxazine tautomers both give a high-quality fit to the powder XRD data in Rietveld refinement, other techniques are required to establish the tautomeric form in the solid state. In particular, high-resolution solid-state ^15^N NMR data support the presence of the alloxazine tautomer, based on comparison between isotropic chemical shifts in the experimental ^15^N NMR spectrum and the corresponding values calculated for the crystal structures containing the alloxazine and isoalloxazine tautomers. Furthermore, periodic DFT-D calculations at the PBE0-MBD level indicate that the crystal structure containing the alloxazine tautomer has significantly lower energy. We also report computational investigations of the interconversion between the tautomeric forms in the crystal structure via proton transfer along two intermolecular N–H···N hydrogen bonds; DFT-D calculations at the PBE0-MBD level indicate that the tautomeric interconversion is associated with a lower energy transition state for a mechanism involving concerted (rather than sequential) proton transfer along the two hydrogen bonds. However, based on the relative energies of the crystal structures containing the alloxazine and isoalloxazine tautomers, it is estimated that under conditions of thermal equilibrium at ambient temperature, more than 99.9% of the molecules in the crystal structure will exist as the alloxazine tautomer.

## Abstract 2

The solid-state structural properties of alloxazine have been determined directly from powder X-ray diffraction data, utilizing high-resolution solid-state ^15^N NMR data and DFT-D calculations to definitively establish that the tautomeric form present in the crystal structure is alloxazine rather than isoalloxazine. Mechanisms for tautomeric interconversion within the crystal structure, via intermolecular proton transfer, have also been explored using DFT-D methodology.

## Introduction

**Figure 1:**
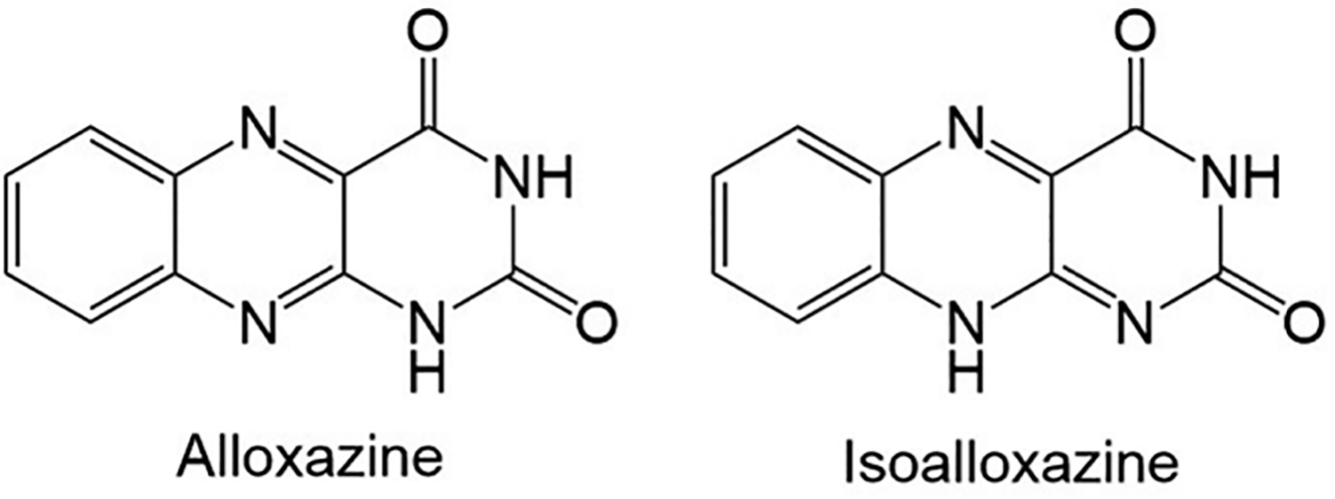
Molecular structures of alloxazine (left) and isoalloxazine (right).

The heterocyclic alloxazine/isoalloxazine ring system is found in many biologically important molecules. For example, it is present in all flavins, such as riboflavin, flavin mononucleotide (FMN), and flavin adenine dinucleotide (FAD); both FMN and FAD are crucial coenzymes for redox processes in a wide range of biological systems.^1^ The alloxazine molecule (Figure 1) may exist in two tautomeric forms, referred to as alloxazine and isoalloxazine, which differ in the position of one hydrogen atom. Recently, alloxazine has been shown to have potential in battery technology as an energy storage material, with functionalization of alloxazine by electron-donating groups leading to improved performance in this application.^2^ To date, the structural properties of alloxazine in the solid state have not been reported; clearly, knowledge of the crystal structure of alloxazine and the preferred modes of intermolecular interactions may yield important insights in understanding the structural chemistry of flavins in different environments.

Although single-crystal X-ray diffraction (XRD) is the most powerful and routine method for crystal structure determination, this technique relies on the availability of a single-crystal specimen of suitable size and quality. When a suitable single crystal cannot be prepared, it is necessary instead to use powder XRD data for structure determination. Although structure determination from powder XRD data is more challenging than from single-crystal XRD data, modern methodology^3−11^ allows crystal structures of organic materials of moderate complexity to be determined directly from powder XRD data, particularly by exploiting the direct-space strategy for structure solution and the Rietveld method for structure refinement. Furthermore, the process of structure determination from powder XRD data may be enhanced^12−18^ by taking advantage of insights gained from complementary experimental techniques (e.g., solid-state NMR spectroscopy) and computational techniques (e.g., periodic DFT-D calculations).

Herein, we report the solid-state structural properties of alloxazine, determined from powder XRD data in conjunction with insights derived from solid-state NMR spectroscopy and DFT-D calculations, which provide important independent evidence allowing the assignment of the tautomeric form (alloxazine versus isoalloxazine) that exists in the crystal structure. Energetic and mechanistic aspects of pathways for interconversion between the alloxazine and isoalloxazine tautomers in the crystal structure are also explored using computational approaches.

## Structure Determination from Powder XRD Data

**Figure 2:**
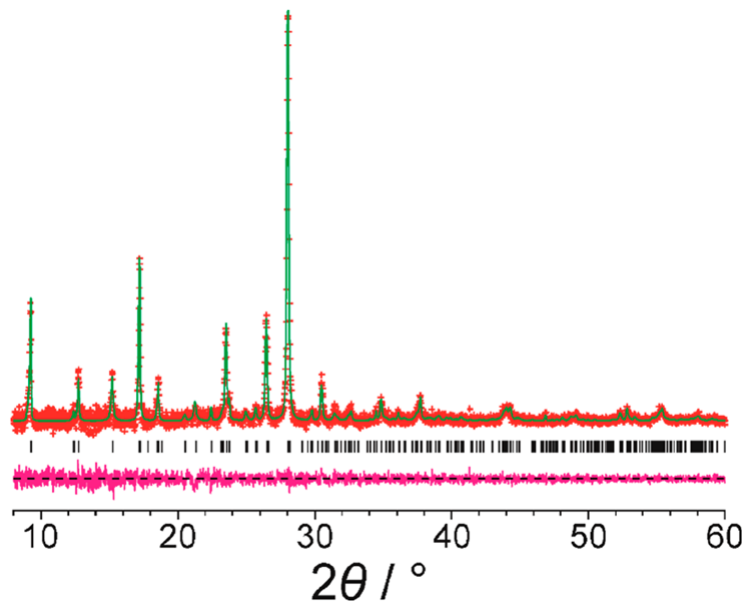
Profile fitting (using the Le Bail method) of the powder XRD data (background subtracted) recorded for alloxazine, showing the experimental powder XRD data (red “+” marks), the calculated powder XRD data (green line), the predicted peak positions (black tick marks), and the difference between experimental and calculated powder XRD data (magenta line).

High-quality powder XRD data were recorded for a sample of alloxazine crystallized from DMSO (see Methods for details). The powder XRD data were indexed using the program LZON^19^ in the CRYSFIRE package,^20^ giving the following unit cell with triclinic metric symmetry: *a* = 5.86 Å, *b* = 7.61 Å, *c* = 10.16 Å, α = 69.9°, β = 83.5°, γ = 83.1° (*V* = 421.2 Å^3^). Unit cell and profile refinement were carried out using the Le Bail method^21^ in the GSAS program package,^22^ giving a good quality of fit to the experimental powder XRD data (Figure 2; *R*_wp_ = 1.07%, *R*_p_ = 0.80%). Knowledge of the unit cell volume and consideration of density suggest that the unit cell contains two molecules of alloxazine. Thus, the asymmetric unit would comprise one molecule (*Z*′ = 1) for space group *P*1 or two molecules (*Z*′ = 2) for space group *P*1.

Structure solution was carried out using the direct-space genetic algorithm (GA) technique incorporated in the program EAGER,^23−26^ which has been applied to solve the crystal structures of materials from a range of areas of application, including materials of biological relevance,^27−29^ framework structures,^30,31^ products from solid-state reactions,^32,33^ optoelectronic materials,^34,35^ multicomponent organic materials,^36,37^ and polymorphic systems.^38^ Structure solution was carried out initially for space group *P*1 (*Z*′ = 1), using a molecular model for alloxazine constructed with standard bond lengths and bond angles from the Cambridge Structural Database using the MOGUL program^39^ and with bond lengths involving hydrogen atoms taken from Allen et al.^40^ As there is one molecule of alloxazine in the asymmetric unit for space group *P*1, each trial structure (using the alloxazine tautomer) was defined by six structural variables (three positional and three orientational variables). In total, 40 independent GA structure-solution calculations were carried out, each starting from a different randomly generated initial population. Each GA calculation involved the evolution of a population of 100 trial structures for 100 generations, with 10 mating operations and 50 mutation operations per generation. All 40 independent calculations produced the same trial structure giving the best fit to the experimental powder XRD data (i.e., lowest *R*_wp_). This structure was considered reasonable on the basis of structural and chemical criteria and was used as the starting structural model for Rietveld refinement.

Structure solution was also carried out for space group *P*1 with *Z*′ = 2. In this case, each trial structure was defined by nine variables (three positional variables and six orientational variables; for space group *P*1, the position of one molecule in the unit cell can be fixed arbitrarily). The trial structure giving the best fit to the experimental powder XRD data was essentially identical to the structure obtained for space group *P*1, confirming that the description of the structure in space group *P*1 is correct.

**Figure 3:**
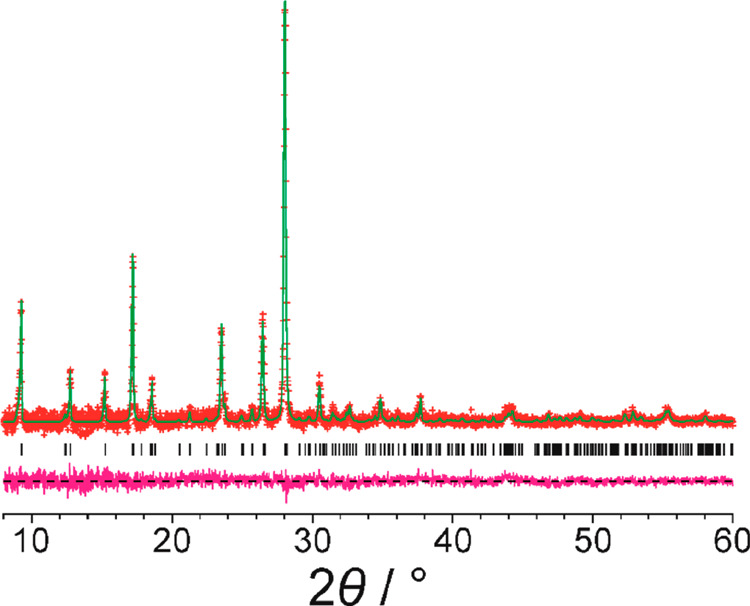
Final Rietveld refinement of the powder XRD data (background subtracted) recorded for alloxazine, showing the experimental powder XRD data (red “+” marks), the calculated powder XRD data (green line), the predicted peak positions (black tick marks), and the difference between experimental and calculated powder XRD data (magenta line).

The best trial structure obtained in the GA structure solution calculations for space group *P*1 was used as the initial structural model for Rietveld refinement,^41^ which was carried out using the GSAS program.^22^ In the Rietveld refinement, standard restraints were applied to bond lengths and bond angles, and planar restraints were applied to the tricyclic ring system. A common isotropic displacement parameter was refined for all non-hydrogen atoms, with the value for hydrogen atoms set to 1.2 times the refined value for the non-hydrogen atoms. No corrections for preferred orientation were applied.^42^ The Rietveld refinement gave an excellent fit to the experimental powder XRD data (Figure 3; *R*_wp_ = 1.15%, *R*_p_ = 0.86%), comparable to the quality of fit obtained in the Le Bail profile fitting (Figure 2), with the following final refined unit cell parameters: *a* = 5.8638(6) Å, *b* = 7.6066(6) Å, *c* = 10.1644(9) Å, α = 69.972(7)°, β = 83.493(8)°, γ = 83.061(6)°, *V* = 421.57(9) Å^3^. The crystal structure from this Rietveld refinement is deposited in the CSD (deposition number: 2111430) and in the Supporting Information.

For structure validation, the final refined crystal structure was subjected to geometry optimization (with fixed unit cell) using periodic DFT-D calculations. The geometry optimized structure shows only minor differences in atomic positions compared to the structure obtained in the Rietveld refinement [with root-mean squared deviation (RMSD) in the positions of the non-hydrogen atoms of 0.082 Å], confirming that the final refined crystal structure is close to a minimum on the energy landscape. The crystal structure from the final Rietveld refinement and the structure obtained after subjecting this structure to DFT-D geometry optimization are compared in Figure S1.

## Results and Discussion

### Crystal Structure of Alloxazine

**Figure 4:**
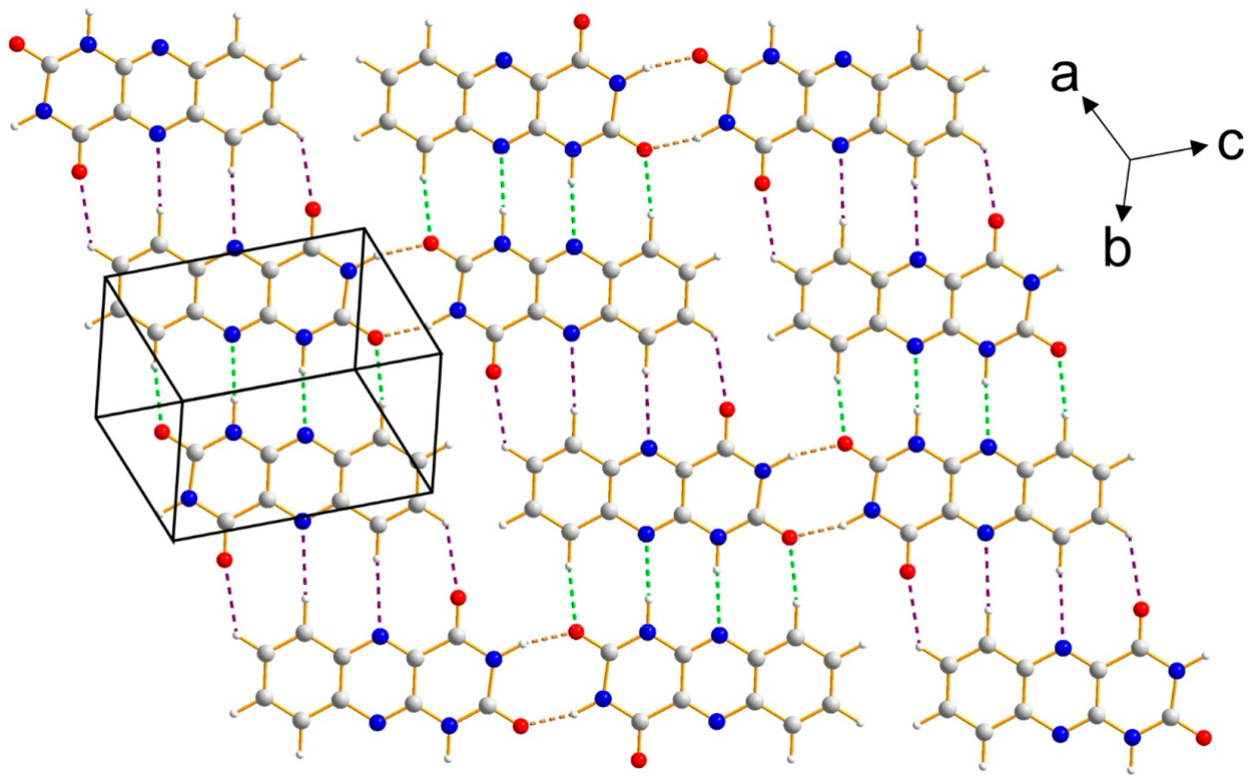
A single hydrogen-bonded layer in the crystal structure of alloxazine viewed perpendicular to the average plane of the layer. Hydrogen bonds (including C–H···N and C–H···O interactions) are indicated by dashed lines with the following color code: A-B interaction motif, green; A-C interaction motif, gold; A-D interaction motif, purple. The A-B, A-C and A-D interaction motifs are defined in Figure 5.

**Figure 5:**
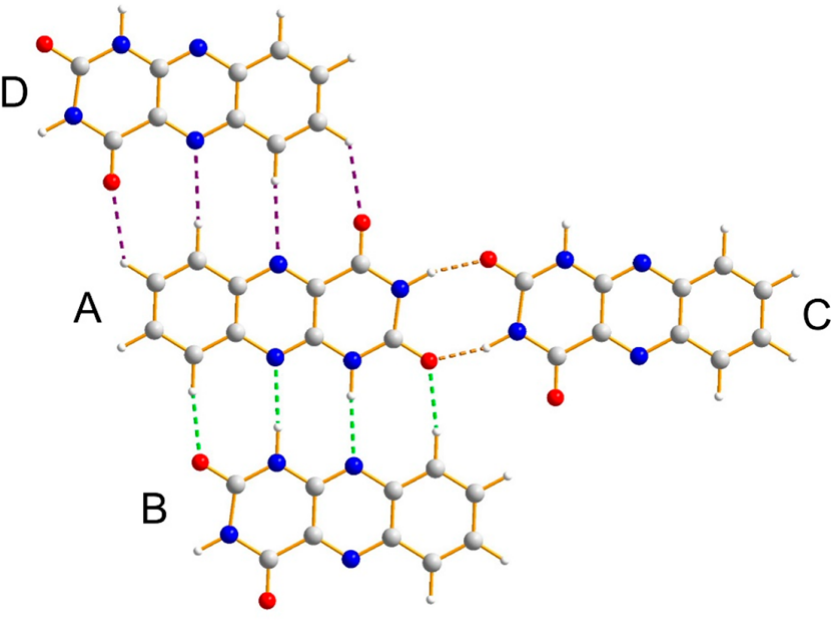
Intermolecular interaction motifs in the crystal structure of alloxazine viewed perpendicular to the molecular planes. Molecules A, B, and D lie very close to the same plane, whereas molecule C is slightly offset in the direction perpendicular to this plane. Hydrogen bonds (including C–H···N and C–H···O interactions) are indicated by dashed lines with the following color code: A-B interaction motif, green; A-C interaction motif, gold; A-D interaction motif, purple.

The crystal structure of alloxazine comprises layers (Figure 4) of molecules connected by three different hydrogen-bonding motifs between a given molecule and three neighboring molecules. Figure 5 shows the intermolecular interactions between a reference molecule, labeled A, and three neighboring molecules within the layer, labeled B, C, and D. The hydrogen bonding to one neighbor (the A-B interaction motif, indicated by green dashed lines in Figures 4 and 5) is a self-complementary motif involving two N–H···N interactions [N···N, 3.110(3) Å; N–H···N, 173.7(3)°] and two C–H···O interactions [C···O, 3.175(7) Å; C–H···O, 170.6(3)°]. The two molecules involved in this interaction motif are related across an inversion center; thus, the two N–H···N interactions are symmetry equivalent, and the two C–H···O interactions are symmetry equivalent. The planes of the two molecules are parallel to each other and lie very close to the same plane.

The interaction with another neighboring molecule in the same layer (the A-D interaction motif, indicated by purple dashed lines in Figures 4 and 5) comprises a set of two C–H···O interactions and two C–H···N interactions. The two molecules involved in this interaction motif are very close to coplanar and are related across an inversion center; thus, the two C–H···O interactions are symmetry equivalent, and the two C–H···N interactions are symmetry equivalent. Based on geometric criteria,^43−45^ these C–H···O interactions [C···O, 3.235(8) Å; C–H···O, 131.0(7)°] and C–H···N interactions [C···N, 3.543(6) Å; C–H···N, 173.3(7)°] are on the borderline of being classified as C–H···O and C–H···N “hydrogen bonds”. For this reason, this set of two C–H···O interactions and two C–H···N interactions is less significant than the A-B interaction motif containing the two strong N–H···N hydrogen bonds discussed above. The combination of the A-B and A-D interaction motifs generates a one-dimensional ribbon of molecules along the [210] direction (close to vertical in Figure 4), with all molecules in the ribbon lying very close to the same plane.

**Figure 6:**
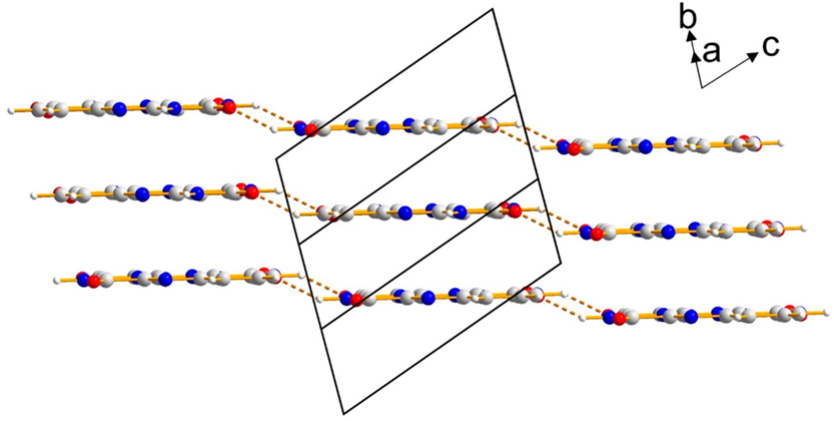
Crystal structure of alloxazine, viewed parallel to the molecular planes, showing three layers of molecules constructed from hydrogen-bonded ribbons. Molecules linked by two N–H···O hydrogen bonds (gold dashed lines) representing the A-C interaction motif (see Figure 5) are not coplanar and are offset slightly in the direction perpendicular to the molecular plane, giving rise to the stepped arrangement shown.

The hydrogen bonding to another neighboring molecule (the A-C interaction motif, indicated by gold dashed lines in Figures 4 and 5) is a cyclic array, denoted  in graph set notation,^46^ constructed from two N–H···O hydrogen bonds [N···O, 2.857(5) Å; N–H···O, 147.6(6)°]. This interaction motif links adjacent ribbons within the hydrogen-bonded layer. The two molecules in this interaction motif are related across an inversion center; however, while the planes of the two molecules are parallel, they are not coplanar, as the positions of the two molecules are slightly offset along the direction perpendicular to the molecular planes (thus, the inversion center lies midway between the planes of the two molecules). As a consequence, the hydrogen-bonded layers are “step-like” rather than flat (see Figure 6), with each step corresponding to the hydrogen-bonded “interface” (comprising A-C interactions) between adjacent ribbons. In Figure 6, the stacking of adjacent hydrogen-bonded layers is viewed parallel to the molecular plane, clearly showing the step-like character of each layer. The perpendicular distance between adjacent layers [taking the mean plane of the stepped layers as (122)] is 3.17 Å; the perpendicular distance between the mean planes of the molecular ribbons in adjacent layers is 3.26 Å.

Finally, we consider the relation between this crystal structure and the crystal structures of derivatives of alloxazine reported previously (CSD Refcodes: CODYEZ10,^47^ FUDWOQ,^48^ FISLID,^49^ AGEPEK^50^), focusing only on derivatives that retain all the hydrogen-bond donor and acceptor groups of the alloxazine molecule (and thus involve only substitution of the C_6_ ring). Among these four structures, all of which are solvates, only the methanol solvate of 7,8-dimethylalloxazine^49^ contains hydrogen-bonding motifs that resemble those in the structure of alloxazine reported here. Specifically, a given molecule of 7,8-dimethylalloxazine is linked to two adjacent molecules by the A-B and A-C interaction motifs, while the other N acceptor is engaged in an O–H···N hydrogen bond with the O–H bond of the methanol molecule (rather than forming the A-D interaction motif observed in the structure of alloxazine). In the other structures, the intermolecular interactions predominantly^47,48^ or entirely^50^ involve hydrogen bonding between the alloxazine derivative and solvent molecules.

### Assessment of Alloxazine/Isoalloxazine Tautomerism

As the alloxazine and isoalloxazine tautomers differ only in the position of one hydrogen atom (with only small geometric differences in other parts of the molecule resulting from the different position of this hydrogen atom), the isoalloxazine tautomer should be able to form an essentially identical crystal structure to alloxazine, with the only significant difference being the position of the hydrogen atom within each intermolecular N–H···N hydrogen bond (i.e., N–H···N versus N···H–N) that links pairs of neighboring molecules (within the A-B interaction motif). Clearly, as the only significant difference between the crystal structures containing the alloxazine and isoalloxazine tautomers would be the position of one hydrogen atom in the asymmetric unit (with only minor structural differences in other aspects of molecular geometry), the powder XRD patterns of the two structures would be virtually indistinguishable.

**Figure 7:**
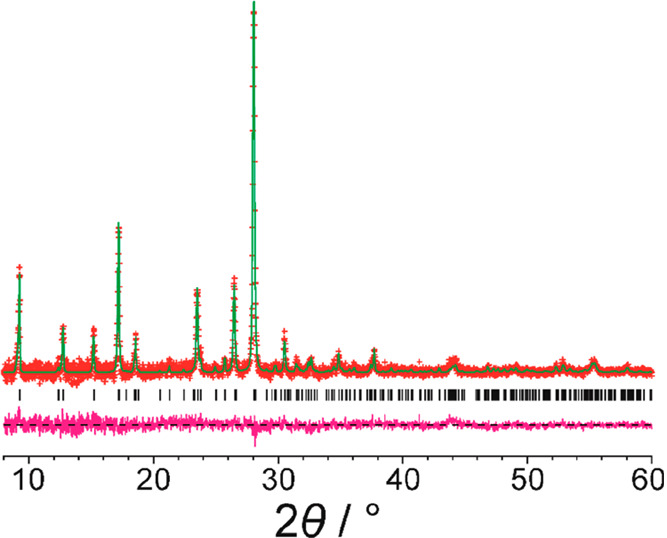
Final Rietveld refinement of the powder XRD data (background subtracted) recorded for alloxazine, using a structural model in which the alloxazine molecule was replaced by the isoalloxazine tautomer. The plot shows the experimental powder XRD data (red “+” marks), the calculated powder XRD data (green line), the predicted peak positions (black tick marks), and the difference between experimental and calculated powder XRD data (magenta line).

To explore this issue in more detail, the molecule in the crystal structure of alloxazine was converted to the isoalloxazine tautomer and then subjected to Rietveld refinement using the same experimental powder XRD data set. The Rietveld refinement gave a good fit to the powder XRD data (Figure 7; *R*_wp_ = 1.18%, *R*_p_ = 0.88%), very similar in quality of fit to the Rietveld refinement using the alloxazine tautomer, although with slightly higher discrepancies between experimental and calculated powder XRD patterns as reflected in the slightly higher values of *R*_wp_ and *R*_p_. The final refined unit cell parameters [*a* = 5.8633(7) Å, *b* = 7.6064(6) Å, *c* = 10.1637(10) Å, α = 69.976(7)°, β = 83.497(9)°, γ = 83.063(6)°, *V* = 421.51(9) Å^3^] are essentially the same, within the estimated standard deviations (esds) in the refined parameters, as those obtained in the Rietveld refinement for alloxazine using the same powder XRD data set. The crystal structure containing the isoalloxazine tautomer from this Rietveld refinement is deposited in the Supporting Information.

The relative energetic stabilities of the crystal structures containing the alloxazine and isoalloxazine tautomers were assessed from periodic DFT-D calculations. Initially, the crystal structure for each tautomer obtained in the final Rietveld refinement was subjected to geometry optimization with fixed unit cell^51^ using the PBE-TS method. In the geometry-optimized crystal structures, the N–H···N hydrogen bond angles are close to linear in each structure (N–H···N: alloxazine, 178.5°; isoalloxazine, 176.4°), but the N–H···N hydrogen bond distances are significantly shorter for isoalloxazine (alloxazine: N···N, 3.09 Å; isoalloxazine: N···N, 2.99 Å). For each geometry-optimized crystal structure, single-point energy calculations were carried out using different functionals (PBE and PBE0) combined with different methods for dispersion correction (TS and MBD). For each calculation method, the calculated energy is *higher* for the isoalloxazine structure than the alloxazine structure, with the following energy differences (expressed per mole of molecules): 17.8 kJ mol^–1^ (PBE-TS); 17.2 kJ mol^–1^ (PBE-MBD); 23.8 kJ mol^–1^ (PBE0-TS); 23.3 kJ mol^–1^ (PBE0-MBD). The magnitude of these energy differences suggests that the crystal structure containing the alloxazine tautomer will be strongly favored in the experimental situation. Calculation of the relative Boltzmann factor for the two tautomeric states indicates that, under conditions of thermal equilibrium at 298 K, more than 99.9% of the molecules in the crystal structure would exist as the alloxazine tautomer. The significant difference in energy between the alloxazine and isoalloxazine crystal structures is dominated by the difference in intramolecular energy between the alloxazine and isoalloxazine tautomers, which favors alloxazine by 54.0 kJ mol^–1^ (PBE0-MBD), rather than differences in the energies of intermolecular interactions within the crystal structures, which favors isoalloxazine by 30.7 kJ mol^–1^ (PBE0-MBD). Thus, the intermolecular cohesion is actually weaker in the crystal structure containing the alloxazine tautomer.

To seek direct experimental verification of the tautomeric form present in the crystal structure, high-resolution solid-state ^15^N NMR and ^13^C NMR data were recorded for the same sample of alloxazine used in the powder XRD study.

**Figure 8:**
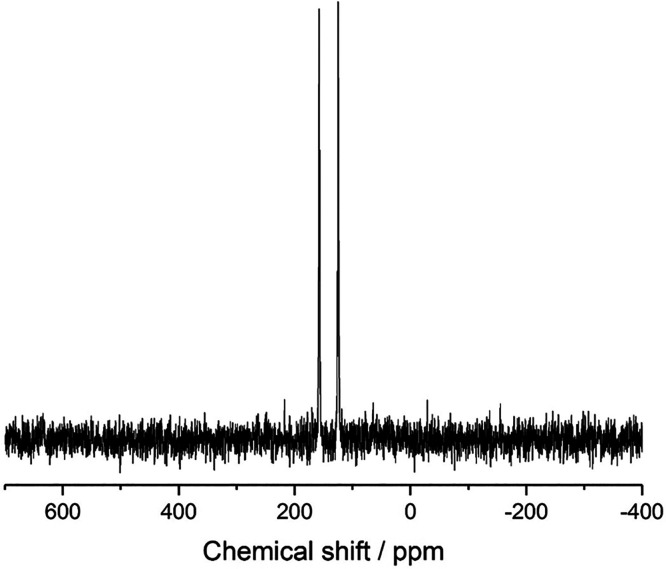
High-resolution solid-state ^15^N NMR spectrum recorded for alloxazine.

The high-resolution solid-state ^15^N NMR spectrum, shown in Figure 8, contains isotropic peaks at 123.81 and 156.56 ppm (chemical shift difference, 32.75 ppm). Although there are four distinct ^15^N environments in the crystal structure (i.e., four distinct ^15^N environments in the alloxazine molecule, with one independent molecule in the asymmetric unit), the conditions of the measurement of the solid-state ^15^N NMR spectrum (involving ^1^H → ^15^N cross-polarization with contact time τ_cp_ = 2 ms) are such that signals of significant intensity are observed only for ^15^N nuclei directly bonded to ^1^H nuclei. Interpretation of the experimental solid-state ^15^N NMR spectrum is greatly facilitated by the capability to calculate the isotropic ^15^N chemical shifts for the crystal structures of the alloxazine and isoalloxazine tautomers determined from the powder XRD data, giving resonances at 126.68 and 161.43 ppm (chemical shift difference, 34.75 ppm) for the two N–H environments in the alloxazine crystal structure (N atoms labeled 2 and 3 in Table 1) and at 149.94 and 165.64 ppm (chemical shift difference, 15.70 ppm) for the two N–H environments in the isoalloxazine crystal structure (N atoms labeled 1 and 3 in Table 1). As shown in Table 1, the match between the calculated and experimental values of the isotropic ^15^N chemical shifts is significantly better for the crystal structure containing alloxazine than the crystal structure containing isoalloxazine. Correspondingly, the chemical shift difference between the two isotropic peaks in the experimental solid-state ^15^N NMR spectrum (32.75 ppm) is in significantly better agreement with the difference between the calculated ^15^N chemical shifts for the two N–H environments in the crystal structure containing alloxazine (34.75 ppm) than the crystal structure containing isoalloxazine (15.70 ppm). These observations give strong support to the conclusion that the material studied in our experimental work contains the alloxazine tautomer rather than the isoalloxazine tautomer.

**Table 1:**
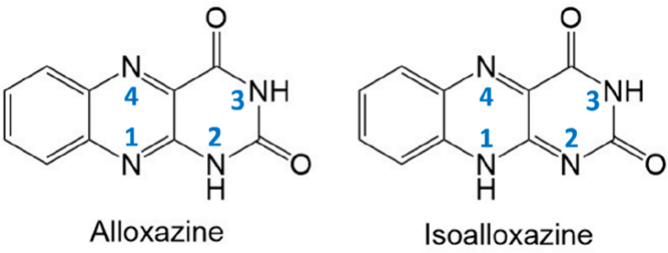
Isotropic ^15^N NMR Chemical Shifts Measured from the Experimental Solid-State ^15^N NMR Spectrum (δ_exp_) and Determined from DFT-D Calculations for the Crystal Structures Containing the Alloxazine Tautomer [δ_calc_(alloxazine)] and the Isoalloxazine Tautomer [δ_calc_(isoalloxazine)]^a^

^15^N site	δ_calc_(alloxazine)/ppm	δ_exp_/ppm	δ_calc_(isoalloxazine)/ppm
1	255.37		**149.94 (NH)**
2	**126.68 (NH)**	123.81 (NH)	182.85
3	**161.43 (NH)**	156.56 (NH)	**165.64 (NH)**
4	337.01		349.36

a The isotropic ^15^N NMR chemical shifts calculated for the NH environments in each crystal structure are highlighted in bold. The numbering of the ^15^N sites in each tautomer is defined in the structures above. Based on comparison between experimental and calculated chemical shifts, the two ^15^N NMR resonances observed in the experimental solid-state ^15^N NMR spectrum are assigned to the NH environments in the crystal structure containing the alloxazine tautomer.

Consideration of the high-resolution solid-state ^13^C NMR spectrum, on the other hand, does not provide a definitive assignment of the tautomeric form. The experimental solid-state ^13^C NMR spectrum contains nine resolved isotropic peaks, which are compared in Figure S2 to the values of the isotropic ^13^C NMR chemical shifts calculated from DFT-D calculations for the crystal structures containing the alloxazine and isoalloxazine tautomers (see also Table S1). Although the overall level of agreement between the experimental and calculated ^13^C NMR data is somewhat better for the crystal structure containing the alloxazine tautomer, the quality of agreement between the experimental and calculated data is not considered to be sufficiently high to lead to a definitive assignment of the tautomeric form based on these results.

### Computational Study of Tautomeric Interconversion in the Crystal Structure of Alloxazine

Another interesting issue relating to the crystal structure of alloxazine concerns the feasibility of interconversion between the alloxazine and isoalloxazine tautomers in the crystal structure, which is considered most likely to occur by proton transfer along the two N–H···N hydrogen bonds that link pairs of neighboring molecules. To explore energetic and mechanistic aspects of this tautomeric interconversion process, transition state calculations were carried out using a nudged elastic band (NEB) method, with the reaction path between the initial and final structures explored using a “chain” of intermediate structures. In the present work, the initial structure was the crystal structure containing the isoalloxazine tautomer, and the final structure was the crystal structure containing the alloxazine tautomer. Each structure within the “chain” of intermediate structures was subjected to periodic DFT-D geometry optimization, while maintaining equal “spacing” to the adjacent structures on the potential energy landscape, which is achieved using a coupling spring constant. A surrogate machine-learned atomistic landscape was used to accelerate convergence (MLNEB), with the efficiency of the transition state search favorably decoupled from the length of the chain representing the reaction path.^52^

**Figure 9:**
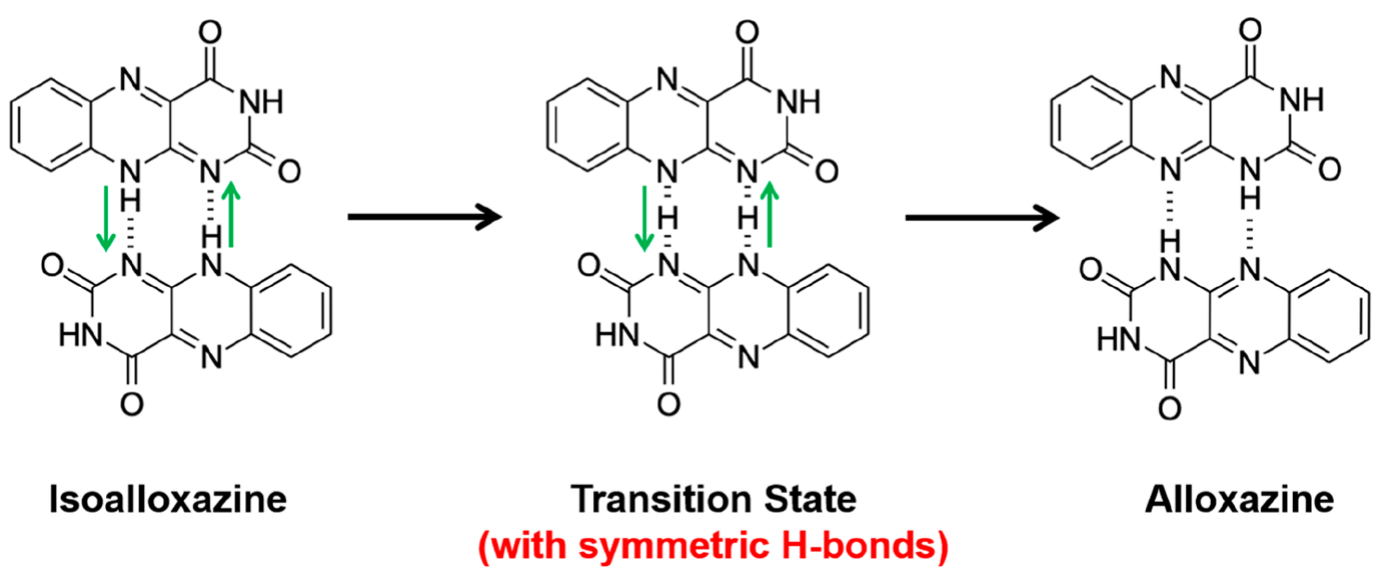
Schematic of the transformation from the isoalloxazine crystal structure to the alloxazine crystal structure by the *concerted mechanism*. Green arrows indicate the directions of proton transfer within the N–H···N hydrogen bonds.

By analogy to proton dynamics in similar hydrogen-bonded systems involving double proton transfer processes within a pair of hydrogen bonds (for example, the carboxylic acid dimer^53−55^), the interconversion between the alloxazine and isoalloxazine tautomers may be envisaged to proceed via a concerted mechanism in which the protons in the two N–H···N hydrogen bonds linking a pair of adjacent molecules transfer *simultaneously* (i.e., N–H···N ↔ N···H–N interconversion) to the other molecule, as shown in Figure 9. This *concerted mechanism* converts both molecules from one tautomer to the other tautomer via a transition state that contains two essentially symmetrical N···H···N hydrogen bonds.

**Figure 10:**
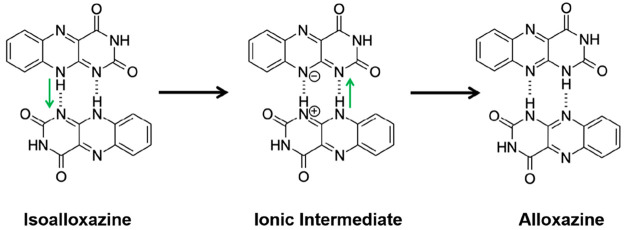
Schematic of the transformation from the isoalloxazine crystal structure to the alloxazine crystal structure by the *sequential mechanism*. Green arrows indicate the directions of proton transfer within the N–H···N hydrogen bonds.

However, our DFT-D studies of the alloxazine/isoalloxazine system also revealed another pathway involving an ionic intermediate. We refer to this mechanism for tautomeric interconversion as the *sequential mechanism*, and it is shown schematically in Figure 10. The sequential mechanism involves two steps (described here in the context of the conversion from isoalloxazine to alloxazine). The first step involves proton transfer along only one of the N–H···N hydrogen bonds between a pair of isoalloxazine molecules, leading to the formation of an ionic intermediate containing a protonated cation and a deprotonated anion, which are linked by two N–H···N hydrogen bonds. The second step involves proton transfer along the other N–H···N hydrogen bond, giving rise to two molecules of the alloxazine tautomer. First, we note that DFT-D geometry optimization carried out (using PBE-TS and fixed unit cell) on the ionic intermediate confirms that it is a local energy minimum, representing a metastable structure on the tautomeric transformation pathway [The crystal structures (after DFT-D geometry optimization using PBE-TS) of alloxazine, isoalloxazine, and the ionic intermediate, as used in our studies of interconversion between the alloxazine and isoalloxazine tautomers, are deposited in the Supporting Information.]. Following geometry optimization of the ionic intermediate (using the PBE-TS method), the energy of this crystal structure was calculated using different methods. In each case, the energy of the ionic intermediate is higher than the energy of the isoalloxazine crystal structure by 9.2 kJ mol^–1^ (PBE-TS), 8.6 kJ mol^–1^ (PBE-MBD), 9.9 kJ mol^–1^ (PBE0-TS), and 9.5 kJ mol^–1^ (PBE0-MBD). The two N–H···N hydrogen bonds linking the anion–cation pair in the ionic intermediate are shorter (N···N, 2.86, 2.91 Å) than those linking pairs of molecules in the crystal structures of alloxazine (N···N, 3.09 Å) and isoalloxazine (N···N, 2.99 Å). Clearly, the inversion center that relates the two N–H···N hydrogen bonds in the crystal structures containing the isoalloxazine and alloxazine tautomers (which have *P*1 space group symmetry) is lost in forming the ionic intermediate, which has *P*1 space group symmetry.

**Figure 11:**
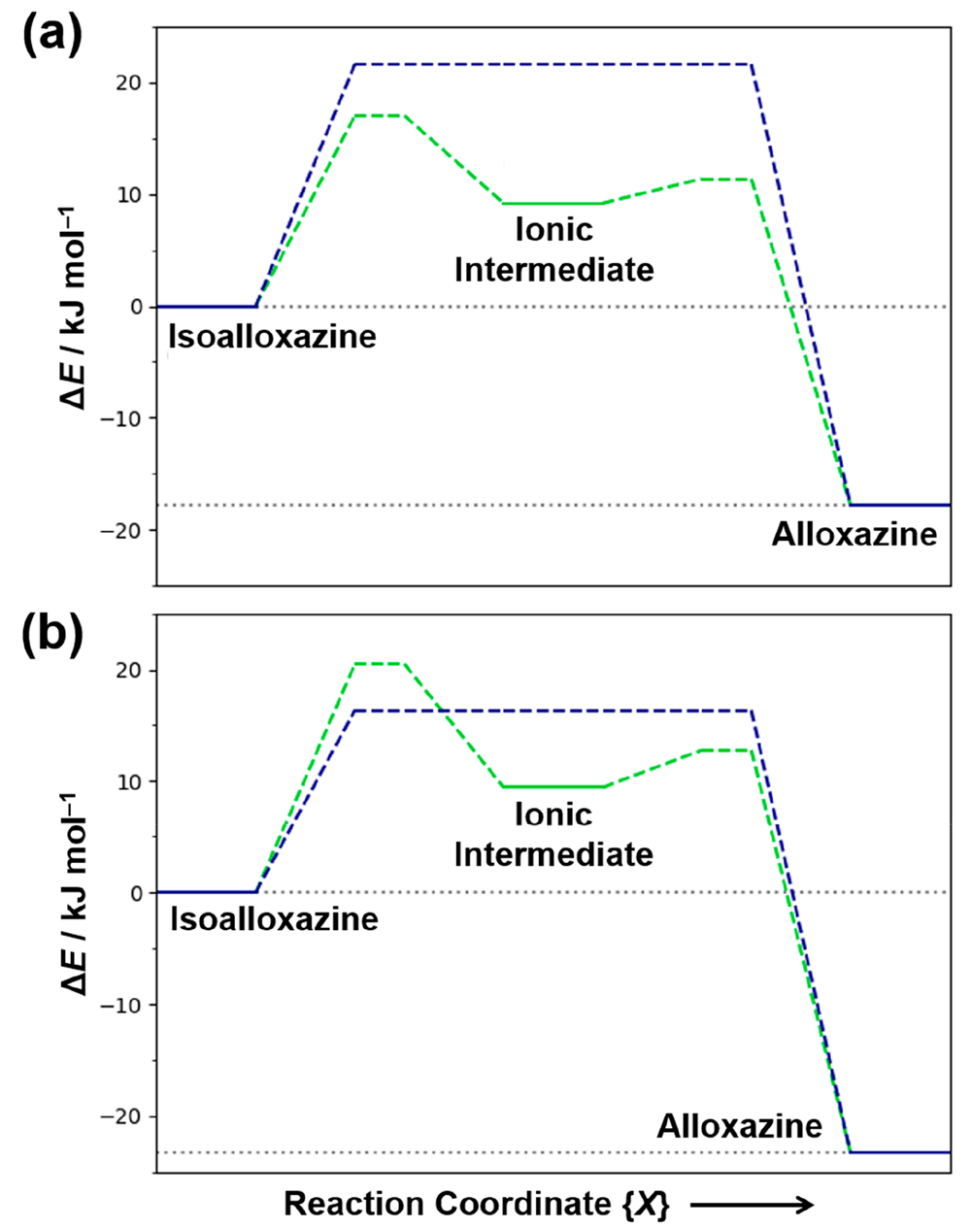
Energy profiles for tautomeric interconversion by the concerted mechanism (blue) and the sequential mechanism (green) calculated using (a) PBE-TS and (b) PBE0-MBD. In each case, the horizontal axis represents the general reaction coordinate, and the vertical axis shows the energy relative to the crystal structure containing the isoalloxazine tautomer. Solid lines indicate stable/metastable structures, dashed lines indicate transition processes (with *horizontal* dashed lines representing transition states), and gray dotted lines indicate the energy levels for the crystal structures containing the alloxazine and isoalloxazine tautomers. For the energy profiles calculated using PBE0-MBD, the concerted mechanism is more favorable. For the energy profiles calculated using PBE-TS, the sequential mechanism is more favorable.

For the calculations using the PBE-TS exchange-correlation functional (Figure 11a), the energy barrier for conversion of the isoalloxazine crystal structure to the alloxazine crystal structure by the concerted mechanism is 21.6 kJ mol^–1^, while the sequential mechanism has a *lower* energy barrier of 17.0 kJ mol^–1^ to convert the isoalloxazine crystal structure to the ionic intermediate and 2.2 kJ mol^–1^ to convert the ionic intermediate to the alloxazine crystal structure. The corresponding calculations using the PBE-MBD and PBE0-TS methods also suggest that the highest energy barrier for the sequential mechanism (PBE-MBD, 15.8 kJ mol^–1^; PBE0-TS, 21.5 kJ mol^–1^), representing the barrier to convert the isoalloxazine crystal structure to the ionic intermediate, is *lower* than the energy barrier for the concerted mechanism (PBE-MBD, 20.0 kJ mol^–1^; PBE0-TS, 28.4 kJ mol^–1^). In contrast, however, the calculations using the PBE0-MBD exchange-correlation functional (Figure 11b) give a *higher* energy barrier for the sequential mechanism (20.5 kJ mol^–1^) than the concerted mechanism (16.3 kJ mol^–1^), indicating that the concerted mechanism is now the favored pathway for the tautomeric transformation at this level of DFT-D computation.

Considering the individual contributions to the total energy for each calculation method shows that, for PBE0-MBD, the transition state in the concerted mechanism is particularly stabilized relative to the crystal structures containing the alloxazine and isoalloxazine tautomers (by 24.1 and 23.1 kJ mol^–1^, respectively) as a result of the many-body dispersion corrections.^56^ In the transition state for the concerted mechanism, the hydrogen atom in each N–H···N hydrogen bond is essentially equidistant between the two nitrogen atoms, and these N–H···N hydrogen bonds are considerably shorter (N···N, 2.69 Å) than those in the crystal structures of the alloxazine tautomer, the isoalloxazine tautomer, and the ionic intermediate in the sequential mechanism (N···N in the range 2.86–3.09 Å; the specific values are discussed above). These shorter N–H···N hydrogen bonds may facilitate the stabilization of the transition state when the many-body dispersion correction is implemented and may explain the lower overall energy barrier calculated using PBE0-MBD. These observations highlight the importance of considering high-level dispersion corrections in future investigations of other proton transfer processes in hydrogen bonds. We note that, among these methods, PBE0-MBD is considered^57,58^ to give the most reliable assessment of the energetic properties of organic materials.

## Concluding Remarks

The crystal structure of alloxazine has been determined directly from powder XRD data, augmented by information from solid-state NMR data and periodic DFT-D calculations. However, the powder XRD data alone cannot definitively establish the tautomeric form present in the crystal structure, as the only significant difference between the crystal structures containing the alloxazine and isoalloxazine tautomers concerns the position of one hydrogen atom in the asymmetric unit (corresponding to the hydrogen atom in each intermolecular N–H···N hydrogen bond). To resolve this issue, further analysis based on periodic DFT-D calculations and solid-state ^15^N NMR spectroscopy strongly support the conclusion that the crystal structure that exists in the experimental situation comprises essentially only the alloxazine tautomer, with no significant population of the isoalloxazine tautomer. These results and conclusions demonstrate the advantages of incorporating insights from spectroscopic data and computational techniques within the protocols for crystal structure determination from powder XRD data.

Computational investigations of the interconversion between the two tautomeric forms of the crystal structure suggest that the sequential mechanism (involving a metastable ionic intermediate) is favored based on periodic DFT-D calculations at the PBE-TS, PBE-MBD, and PBE0-TS levels, whereas the concerted mechanism (involving simultaneous transfer of protons in two N–H···N hydrogen bonds between pairs of molecules) is favored based on periodic DFT-D calculations at the PBE0-MBD level. Given that the PBE0-MBD method is widely accepted as giving the most reliable assessment of the energetic properties of organic materials, this observation has wider implications concerning the importance of using high-level dispersion corrections in computational studies of structural transformations in crystalline solids based on mechanisms involving proton transfer.

## Methods

### Sample Preparation

Alloxazine was purchased from Sigma-Aldrich as a fine, yellow powder. The material was recrystallized by cooling a solution of alloxazine in DMSO from 130 °C to ambient temperature. The powder XRD patterns of the directly purchased material and the recrystallized material were identical, representing the same solid phase of alloxazine. No other crystallization solvents or crystallization methods were investigated.

### Powder XRD

To record high-quality powder XRD data for structure determination, a sample of the recrystallized material was lightly ground in a mortar and pestle and then loaded into a 0.7 mm glass capillary, which was flame-sealed. The powder XRD data were recorded at 21 °C on a Bruker D8 diffractometer operating in transmission mode using Ge-monochromated CuKα_1_ radiation (2θ range, 4°–70°; step size, 0.016°; data collection time, 15.5 h).

### Solid-State NMR Spectroscopy

High-resolution solid-state ^13^C NMR data were recorded on a Bruker AVANCE III HD spectrometer (magnetic field, 9.4 T; ^13^C Larmor frequency, 100.64 MHz), with the powder sample packed in a zirconia solid-state NMR rotor. The ^13^C NMR spectrum was recorded at ambient temperature using ramped ^1^H → ^13^C cross-polarization (CP), magic-angle spinning (MAS spinning frequency, 10 kHz), and ^1^H decoupling using the SPINAL-64 sequence.^59^ The ^13^C NMR spectrum was referenced^60^ against the α polymorph of glycine, with the carboxylate resonance set to 176.5 ppm.

High-resolution solid-state ^15^N NMR data were recorded at 20.0 T on a Bruker AVANCE III spectrometer at the U.K. High-Field (850 MHz) Solid-State NMR Facility (^15^N Larmor frequency, 86.16 MHz; ^1^H Larmor frequency, 850.24 MHz) with a 4 mm HY MAS probe (MAS frequency, 8 kHz) using ramped ^1^H → ^15^N cross-polarization (CP contact time, 2 ms). The ^15^N NMR spectrum was referenced^61^ using the isotropic ^15^N NMR chemical shift for the δ-^15^N site in histidine hydrochloride monohydrate at δ = 189.5 ppm.

### Periodic DFT-D Calculations

Periodic DFT-D calculations were carried out at various stages of this work. In conjunction with validation of the crystal structure determined from powder XRD data, periodic DFT-D geometry optimization was carried out (with fixed unit cell) using CASTEP^62^ (Academic Release version 8.0). The calculations used ultrasoft pseudopotentials,^63^ PBE functional,^64^ semiempirical dispersion corrections using the TS correction scheme,^65^ preserved space group symmetry, periodic boundary conditions, a basis set cutoff energy of 700 eV, and a Monkhorst–Pack grid^66^ of minimum sample spacing (0.05 × 2π) Å^–1^. The convergence criteria for geometry optimization were 0.01 eV Å^–1^ for atomic forces, 0.00001 eV per atom in the total energy, and 0.001 Å for atomic displacements.

Solid-state NMR chemical shifts were calculated using CASTEP (Academic Release version 8.0) for the crystal structures containing the alloxazine and isoalloxazine tautomers determined from powder XRD data, followed by DFT-D geometry optimization (with fixed unit cell) as described above. The Gauge Including Projector Augmented Wave (GIPAW) approach^67−72^ was used with a cutoff energy of 700 eV and PBE functional. For each of the crystal structures containing the alloxazine and isoalloxazine tautomers, a set of isotropic ^13^C NMR shielding values and a set of isotropic ^15^N NMR shielding values were generated from the CASTEP calculations.

From the isotropic ^13^C NMR shielding value (σ_calc_) calculated for each ^13^C environment in the crystal structure, the corresponding calculated isotropic ^13^C NMR chemical shift (δ_calc_) was determined^69^ from the equation δ_calc_ = ⟨δ_exp_⟩ + ⟨σ_calc_⟩ – σ_calc_, where ⟨δ_exp_⟩ denotes the mean of the isotropic ^13^C NMR chemical shifts determined from the experimental high-resolution solid-state ^13^C NMR spectrum (⟨δ_exp_⟩ = 140.45 ppm) and ⟨σ_calc_⟩ denotes the mean of the calculated isotropic ^13^C NMR shielding values (⟨σ_calc_⟩ = 30.18 ppm for alloxazine; ⟨σ_calc_⟩ = 30.71 ppm for isoalloxazine).

From the isotropic ^15^N NMR shielding value (σ_calc_) calculated for each ^15^N environment in the crystal structure, the corresponding calculated isotropic ^15^N NMR chemical shift (δ_calc_) was determined from the equation δ_calc_ = δ_exp_(ref) + σ_calc_(ref) – σ_calc_, where δ_exp_(ref) denotes the experimental value of the isotropic ^15^N NMR chemical shift [δ_exp_(ref) = 189.5 ppm] for the reference material (the δ-^15^N site in histidine hydrochloride monohydrate) and σ_calc_(ref) denotes the isotropic ^15^N NMR shielding value [σ_calc_(ref) = 29.2 ppm] for the δ-^15^N site in histidine hydrochloride monohydrate calculated for the known crystal structure of this material^73^ using the same DFT-D methodology as that used to calculate the ^15^N shielding values for the alloxazine and isoalloxazine crystal structures.

To determine the relative energies of the crystal structures containing the alloxazine and isoalloxazine tautomers and to assess the energy barriers to convert between these crystal structures, DFT-D calculations were carried out using FHI-aims^74^ (date stamp: 191029) coupled with the Atomic Simulation Environment (ASE) package^75^ and the Catlearn Python package.^76^ Geometry optimization was carried out using the BFGS algorithm implemented in FHI-aims, with a convergence criterion of 0.01 eV Å^–1^ for atomic forces. Transition states were determined using the machine-learning nudged-elastic band (MLNEB) algorithm provided by Catlearn,^52^ with a convergence criterion of 0.05 eV Å^–1^ for atomic forces. In both types of calculation, the PBE version^64^ of the general gradient approximation (GGA) was coupled with the Tkatchenko-Scheffler (TS) dispersion correction.^65^ Following convergence of the geometry optimization procedures, single-point energy calculations were carried out using both GGA and hybrid-GGA exchange-correlation functionals, specifically PBE^64^ and PBE0,^77^ coupled with either the TS method^65^ or the many-body dispersion (MBD) method^78^ for dispersion correction. Thus, the complete set of exchange-correlation functionals considered in this work was as follows: PBE-TS, PBE-MBD, PBE0-TS, and PBE0-MBD. The electronic structure calculations were carried out with an “intermediate” basis set, and relativistic effects were included via the scaled zeroth order regular approximation.^74^ A Γ-centered *k*-grid was used with a minimum sample spacing of (0.05 × 2π) Å^–1^ [Testing with a denser *k*-grid sampling of (0.04 × 2π) Å^–1^ gave changes in relative energies less than 1 meV.]. The electronic structure self-consistent field (SCF) cycle was considered to be converged when changes in the electron density, the total energy, and the sum of the eigenvalue energies were below 10^–6^ e a_0_^–3^, 10^–6^ eV, and 10^–6^ eV, respectively.

Additional supporting experimental data and computational data for this article may be accessed at 10.17035/d.2021.0140049329 and 10.17172/NOMAD/2021.10.15-1, respectively.

## Supporting Information Available

Crystal structures containing the alloxazine and isoalloxazine tautomers obtained from Rietveld refinement; and crystallographic information for crystal structures (following DFT-D geometry optimization using PBE-TS) relevant to DFT-D studies of interconversion between the alloxazine and isoalloxazine tautomers, specifically crystal structures of alloxazine, isoalloxazine, and the ionic intermediate (ZIP)
Comparison of the crystal structure of alloxazine from Rietveld refinement and the crystal structure obtained following DFT-D geometry optimization of this structure; experimental and calculated solid-state ^13^C NMR data (PDF)

## Accession Codes

CCDC 2111430 contains the supplementary crystallographic data for this paper. These data can be obtained free of charge via www.ccdc.cam.ac.uk/data_request/cif, or by emailing data_request@ccdc.cam.ac.uk, or by contacting The Cambridge Crystallographic Data Centre, 12 Union Road, Cambridge CB2 1EZ, UK; fax: +44 1223 336033.
