# Supplementary material for: Solid-State Structural Properties of Alloxazine Determined from Powder XRD Data in Conjunction with DFT-D Calculations and Solid-State NMR Spectroscopy: Unraveling the Tautomeric Identity and Pathways for Tautomeric Interconversion
Source: Cryst Growth Des. 2021 Nov 22;22(1):524–34. doi: 10.1021/acs.cgd.1c01114 (PMC8739831; doi:10.1021/acs.cgd.1c01114)

Supporting Information

Solid-state structural properties of alloxazine determined from powder XRD data in conjunction with DFT-D calculations and solid-state NMR spectroscopy: unravelling the tautomeric identity and pathways for tautomeric interconversion

Christopher J. H. Smalley,[1] Andrew J. Logsdail,[2] Colan E. Hughes,[1] Dinu Iuga,[3] Mark T. Young,[4] Kenneth D. M. Harris[1]*

[1] School of Chemistry, Cardiff University, Park Place, Cardiff, CF10 3AT, Wales, United Kingdom

[2] Cardiff Catalysis Institute, School of Chemistry, Cardiff University, Park Place, Cardiff, CF10 3AT, Wales, United Kingdom

[3] Department of Physics, University of Warwick, Coventry, CV4 7AL, United Kingdom

[4] School of Biosciences, Cardiff University, Cardiff, CF10 3AX, Wales, United Kingdom

* Author for correspondence: HarrisKDM@cardiff.ac.uk

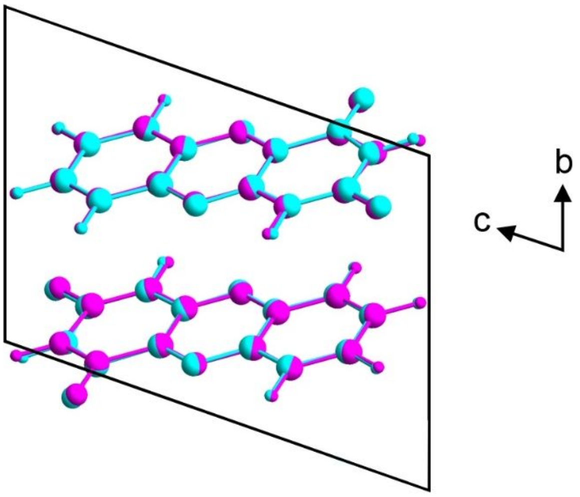

Figure S1. Overlay of the crystal structure of alloxazine from the final Rietveld refinement (magenta) and following DFT-D geometry optimization (with fixed unit cell) of this crystal structure (cyan).

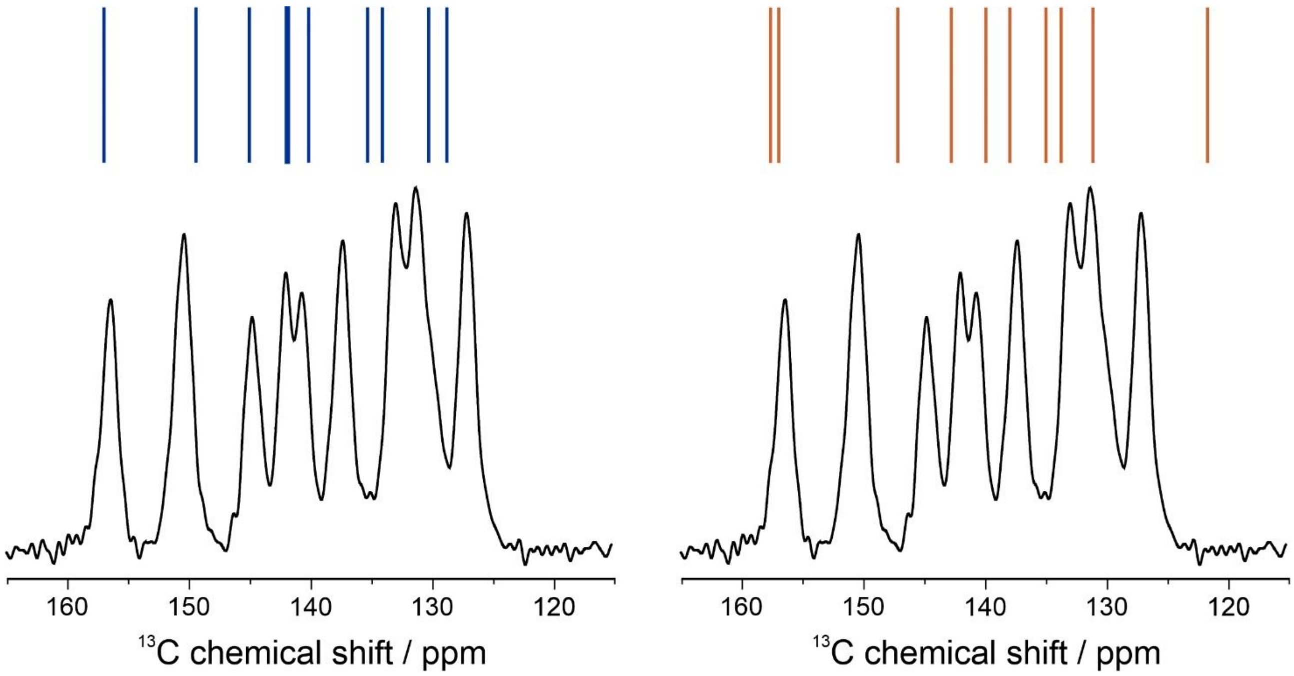

Figure S2. High-resolution solid-state ^{13}C NMR data. Left: the experimental spectrum recorded for the sample of alloxazine studied in this work together with the values of isotropic ^{13}C NMR chemical shifts calculated for the crystal structure containing the alloxazine tautomer (blue lines; the thicker line arises from two calculated isotropic chemical shifts with very similar values). Right: the experimental spectrum together with the values of isotropic ^{13}C NMR chemical shifts calculated for the crystal structure containing the isoalloxazine tautomer (brown lines). The isotropic ^{13}C NMR chemical shifts calculated for each crystal structure are listed in Table S1.

Table S1. Isotropic ^{13}C NMR chemical shifts calculated for the crystal structure containing the alloxazine tautomer and for the crystal structure containing the isoalloxazine tautomer. The numbering of the ^{13}C sites in each tautomer is defined in the figure below.

	δ_{calc} / ppm	
^{13}C site	alloxazine	isoalloxazine
1	145.10	138.01
2	149.48	157.00
3	157.03	157.68
4	130.35	147.23
5	141.97	131.17
6	134.14	121.77
7	135.39	142.82
8	140.23	135.06
9	128.87	133.79
10	142.01	139.99

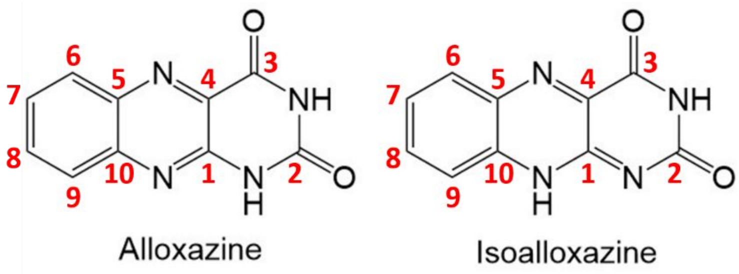

Supplement: Supplementary file 1 — cg1c01114_si_001.pdf [file cg1c01114_si_001.pdf]
